# Lead Induces Similar Gene Expression Changes in Brains of Gestationally Exposed Adult Mice and in Neurons Differentiated from Mouse Embryonic Stem Cells

**DOI:** 10.1371/journal.pone.0080558

**Published:** 2013-11-19

**Authors:** Francisco Javier Sánchez-Martín, Yunxia Fan, Diana M. Lindquist, Ying Xia, Alvaro Puga

**Affiliations:** 1 Department of Environmental Health and Center for Environmental Genetics, University of Cincinnati, College of Medicine, Cincinnati, Ohio, United States of America; 2 Cincinnati Children's Hospital Medical Center, Department of Radiology, Cincinnati, Ohio, United States of America; University of Kentucky, United States of America

## Abstract

Exposure to environmental toxicants during embryonic life causes changes in the expression of developmental genes that may last for a lifetime and adversely affect the exposed individual. Developmental exposure to lead (Pb), an ubiquitous environmental contaminant, causes deficits in cognitive functions and IQ, behavioral effects, and attention deficit hyperactivity disorder (ADHD). Long-term effects observed after early life exposure to Pb include reduction of gray matter, alteration of myelin structure, and increment of criminal behavior in adults. Despite growing research interest, the molecular mechanisms responsible for the effects of lead in the central nervous system are still largely unknown. To study the molecular changes due to Pb exposure during neurodevelopment, we exposed mice to Pb *in utero* and examined the expression of neural markers, neurotrophins, transcription factors and glutamate-related genes in hippocampus, cortex, and thalamus at postnatal day 60. We found that hippocampus was the area where gene expression changes due to Pb exposure were more pronounced. To recapitulate gestational Pb exposure *in vitro*, we differentiated mouse embryonic stem cells (ESC) into neurons and treated ESC-derived neurons with Pb for the length of the differentiation process. These neurons expressed the characteristic neuronal markers *Tubb3*, *Syp*, *Gap43*, *Hud*, *Ngn1*, *Vglut1* (a marker of glutamatergic neurons), and all the glutamate receptor subunits, but not the glial marker *Gafp*. Importantly, several of the changes observed in Pb-exposed mouse brains *in vivo* were also observed in Pb-treated ESC-derived neurons, including those affecting expression of *Ngn1*, *Bdnf* exon IV, *Grin1*, *Grin2D*, *Grik5*, *Gria4*, and *Grm6*. We conclude that our ESC-derived model of toxicant exposure during neural differentiation promises to be a useful model to analyze mechanisms of neurotoxicity induced by Pb and other environmental agents.

## Introduction

Lead, a non-biodegradable metal, is one of the most ubiquitous persistent toxicants present in the environment. Unlike other heavy metals, like iron, copper or manganese, lead has no known biological functions [1], but, because of its physico-chemical properties of high malleability, ductility, softness, low melting point and resistance to corrosion, it has many industrial uses, including manufacturing of pipes, lead-based paints, ceramic glazes, batteries, pottery, and ammunition.

Occupational and accidental lead poisoning have been described since the time of the ancient Romans [2,3]. Pb inhibits detoxification enzymes, heme synthesis, and cholesterol metabolism, causing liver damage, renal dysfunction, hypertension and encephalopathy [4–6], and several reproductive system problems, including reduction of libido, delay in puberty and infertility [3,7,8]. Chronic and low-dose exposure to Pb during prenatal life and early childhood damages the CNS. Solid epidemiological evidence has linked Pb exposure during early childhood to deficits in cognitive functions and IQ, behavioral effects, and attention deficit hyperactivity disorder (ADHD) [9–11]. Importantly, early life exposure to Pb can have remote effects later in life, producing persistent injury in adults, including gray matter volume loss in prefrontal cortex [12,13]. Other long-term consequences of childhood lead exposure include changes of myelin structure in white matter [14] and low level of activation in brain areas associated with language function, such as left frontal cortex and left middle temporal gyrus [15]. Neurochemically, lead decreases the brain concentration of important metabolites, such as N-acetyl aspartate, cholines, creatinine, phosphocreatinine, and a composite of glutamate and glutamine [13]. Behaviorally, blood lead levels during childhood have been correlated to an increase of criminal behavior in adult life [9–11,16]. Altogether, these data suggest that lead exposure during early life may produce irreversible neuronal dysfunction and reorganization that last into adult life. 

There is a significant body of evidence in support of this contention. Twenty-three years after exposure to Pb during infancy, *Macaca fascicularis* monkeys showed elevated expression of Alzheimer disease-related genes, including β-amyloid precursor protein (APP) and β-site APP cleaving enzyme 1 (BACE1), and an increase of total amyloid plaques in the cortex [17]. Significantly, the aging brains of these monkeys that were exposed to lead as infants also showed changes in the epigenetic machinery, with a reduction in total DNA methylation, DNA methyltransferases-1 and -3A, and methyl CpG binding protein-2 levels, and modifications of histone marks critical for the regulation of gene expression [17,18]. Similar results have been observed in the cortical region of rodents, in which early life-Pb exposure altered gene expression patterns and global methylation profiles [19,20], and in zebrafish (*Danio rerio*), where Pb altered neurological development pathways and caused neurotoxicity [21,22]. 

Recent studies using primary neuronal cultures indicate that lead exposure during childhood may negatively modify important neuronal pathways implicated in the late effects observed during adult life, especially affecting pathways implicated in synaptic plasticity, learning, memory, and cell survival, including modification of the N-Methyl-D-Aspartate (NMDA) receptor architecture [23,24], changes of the activity of Ca^2+^/calmodulin dependent protein kinase II (CaMKII), phosphorylation of transcription factor CREB, and expression and translocation of brain-derived neurotrophic factor (BDNF) [25,26]. Pb exposure also represses the expression of presynaptic vesicular proteins implicated in neurotransmitter release, such as synaptobrevin (VAMP1) and synaptophysin (SYN), while it increases p75 neurotrophin receptor (p75(NTR)) levels and alters TrkB-p75(NTR) colocalization in glutamate synapses [26,27]. 

In this study we have initiated a test of the hypothesis that the Pb-induced alterations that occurs during neural development may be responsible for the behavior and cognitive impairment observed in adult life. We exposed mice to Pb *in utero* and found that these mice presented alterations in the expression of several genes, including neural markers, neurotrophins, transcription factors, and glutamate-related genes. We also induced mouse ESCs to differentiate into neurons and treated them with Pb during the differentiation process and observed that some of the changes induced by Pb in the brains of mice were also found *in vitro*. This *in vitro* model may serve as a powerful tool to study the mechanisms of lead and other environmental neurotoxicants.

## Materials and Methods

### Mice and lead exposure

C57BL/6J mice were housed in the vivarium at Cincinnati Children's Hospital Medical Center under controlled conditions of temperature, humidity, and lighting, and provided standard mouse chow and water ad libitum. All experimental procedures conducted with these animals have been approved by the University of Cincinnati and Cincinnati Children's Animal Care and Use Committees. Female mice were exposed to 0 or 3 ppm of lead acetate in drinking water from 8 weeks prior to mating, through gestation and until postnatal day PD10. This treatment regimen insured steady state lead levels in the dam at the time of gestation. From each treatment group, brain tissues of 3 male offspring, including cortex, hippocampus, and thalamus were collected at PD60.

### Mouse embryonic stem cells culture, *in vitro* differentiation and treatments

Undifferentiated C57BL/6-C2 mouse mESC [28] were cultured in ES medium, consisting of Dulbecco’s modified Eagle’s medium (DMEM, Gibco) supplemented with 15% (v/v) ESC qualified fetal bovine serum (knockout serum replacement FBS, Gibco), 2 mM L-glutamine (Gibco), 0.1 mM 2-β-mercaptoethanol (βME, Gibco), 0.1 mM non-essential amino acids (NEAA, Gibco), 1 mM sodium pyruvate (Gibco), 100 U/ml penicillin (Gibco), 100 μg/ml streptomycin (Gibco), and 1000 U/ml leukemia inhibitory factor (LIF, Millipore). Cells were plated in 0.1% (w/v) gelatin-coated dishes at 37°C in a humidified atmosphere with 5% CO_2_, and passaged every two days. Neural differentiation followed protocols previously described by others [29]. Briefly, to obtain cellular aggregates (CA), 4 x 10^6^ undifferentiated mESCs were plated in bacteriological dishes for 8 days using CA medium, consisting of DMEM supplemented with 10% fetal bovine serum (FBS, Gibco), 2 mM L-glutamine, 550 nM βME, 0.1 mM NEAA, 100 U/ml penicillin, and 100 µg/ml streptomycin. On day 4, the CA medium was made to contain 5 μM retinoic acid (Sigma-Aldrich). To obtain a cellular suspension, cells were trypsinized and plated at 2 x 10^5^ cells/cm^2^ in 24-well or 6-well plates coated with poly-DL-ornithine (Sigma-Aldrich) and laminin (Roche), using N2 medium, consisting of a 1:1 mixture of DMEM and DMEM nutrient mixture F-12 ham (Sigma-Aldrich) supplemented with 2 mM L-glutamine, 50 μg/ml BSA, 100 nM putrescine, 20 nM progesterone, 30 nM sodium selenite, 25 μg/ml insulin, 25 μg/ml transferrin, 100 U/ml penicillin, and 100 μg/ml streptomycin. After 48 hours, the N2 medium was replaced with complete medium, consisting of DMEM supplemented with 2 mM L-glutamine, 50 μg/ml BSA, 1X serum-free medium supplement B27 (GIBCO), 30 nM sodium selenite, 25 μg/ml insulin, 25 μg/ml transferrin, 100 U/ml penicillin, and 100 μg/ml streptomycin. The cells were maintained at 37°C in a humidified atmosphere with 5% CO_2_. When needed, cells were treated with lead acetate (Sigma-Aldrich) at different concentrations for the duration of neural differentiation.

### Cell lysates and Western immunoblot analysis

Western immunoblots were performed as previously described [30]. Briefly, cells were lysed by sonic disruption on ice in NETN buffer. Samples were cleared from debris by centrifugation at 14000 rpm for 15 minutes. Protein concentration of the supernatants was determined by the Bradford assay. Tissue samples were homogenized prior to lysis using an Ultra-Turrax T25 homogenizer (Janke& Kunkel, Ika® Labortechnik). Proteins were separated in 8% SDS-polyacrylamide gels and transferred to polyvinylidene fluoride (PVDF) membranes by electroblotting. Immunoblots were probed with antibodies specific for neuronal β3 tubulin (TUBB3) (Abcam), glial fibrillary acidic protein (GFAP) (Millipore) and β-actin (Sigma-Aldrich) for 2 hours at room temperature in TBS-T buffer containing 5% (w/v) non-fat milk. After washing, blots were incubated with the appropriate horseradish peroxidase-modified secondary antibodies in TBS-T for 1 hour at room temperature. Protein bands were visualized by incubation with chemiluminescent PicoWest Super Signal (Pierce) and exposure to X-Ray film. Band intensity was evaluated using ImageJ software.

### Immunofluorescence

Cellular aggregates were dissociated with trypsin and the cells were grown on coverslips at 2 x 10^5^ cells/cm^2^. After 3 days in culture, cells, termed 3-DIV cells for 3-days-*in-vitro*, were fixed with 4% paraformaldehyde for 15 minutes at room temperature, washed twice with PBS and cells were permeabilized and blocked in a blocking solution containing 0.05% (v/v) Triton X-100 and 2% (w/v) BSA in PBS. Primary antibodies were incubated in blocking solution for 2 hours at room temperature at 1:500 for TUBB3, 1:500 for vesicular glutamate transporter 1 (VGLUT1) (Synaptic Systems), and1:1000 for GFAP. Samples were washed twice in a washing solution containing 0.05% (v/v) Triton X-100 in PBS and incubated with the appropriate secondary antibodies in blocking solution at room temperature for 1 hour at 1:100 for Alexa 488 anti-mouse, and 1:100 for Alexa 488 anti-rabbit (Molecular Probes). After the second antibody was removed, samples were washed twice with washing solution and incubated with 2 μg/ml Hoechst in PBS for 10 minutes. Coverslips were mounted onto slides using 44% glycerol in PBS. Micrographs were taken at 40X using an Axioplan Zeiss fluorescent microscope equipped with an AxioCam ERc5s and Zeiss’ Zen Microscopy suite application. 

### Reverse transcription and quantitative real-time RT-PCR

Total RNA was isolated from undifferentiated and differentiated cells by using TRIzol reagent (Ambion) and from tissues by using a RNeasy Minikit (Qiagen). Reverse transcription was performed using random hexamer primers and SuperScript III transcriptase (Invitrogen) as previously described [31]. Quantitative real-time RT-PCR was used to quantify the expression levels of different genes, using *Gapdh* mRNA as the normalization standard. Primers used are shown in Table S1. Raw data is shown as 2 to the power of - ΔΔC_t_, where ΔΔC_*t*_ = (*Ct* ^Gene^ - *Ct* ^Gapdh^)_Assay_.

### Active caspase-3 analysis by flow cytometry

Undifferentiated ESC and cellular aggregates were trypsinized to obtain isolated cells and collected by centrifugation at 200x*g* for 5 minutes. After washing twice with PBS, active caspase-3 was assayed with a FITC Active Caspase-3 Apoptosis Kit (BD Pharmigen) following the manufacturer’s protocols and processed by flow cytometry. 

### Statistical analysis

All differentiation experiments were done using at least three independent cultures. Data are shown as the mean ± SEM. Group comparisons were made using one-way ANOVA followed by post hoc Tukey test. A *p*-value < 0.05 was considered statistically significant.

## Results

### Gene expression changes induced by gestational Pb exposure

Prenatal and early childhood lead exposure are associated with adverse cognitive, neurobehavioral and motor outcomes, suggesting altered brain structure and function and, by extension, altered gene expression patterns in the CNS [14]. These alterations can be extended also to adolescence and adult life as a consequence of peak blood Pb levels above 5-10 µg/dl during childhood [15,32]. To study gene expression changes induced by Pb in the brain of mice, we exposed female mice to 0 or 3 ppm of lead acetate in drinking water from 8 weeks prior to mating, through gestation and until postnatal day PD10, and collected brain tissues of 3 male offspring from each treatment group at PD60. For gene expression analyses we chose cortex, hippocampus, and thalamus because of their implication in learning and memory formation [33–35] and the known loss of gray mass volume in cortex resulting from Pb exposure in humans [36]. Recently, it has been shown that 27 ppm of gestational lead exposure in drinking water produced a concentration of Pb in blood of 10 μg/dl at PN10 in C57BL/6J mice [37]. Therefore, we used one tenth the amount of this metal of what has been considered the action level and human-equivalent of Pb.

**Figure 1:**
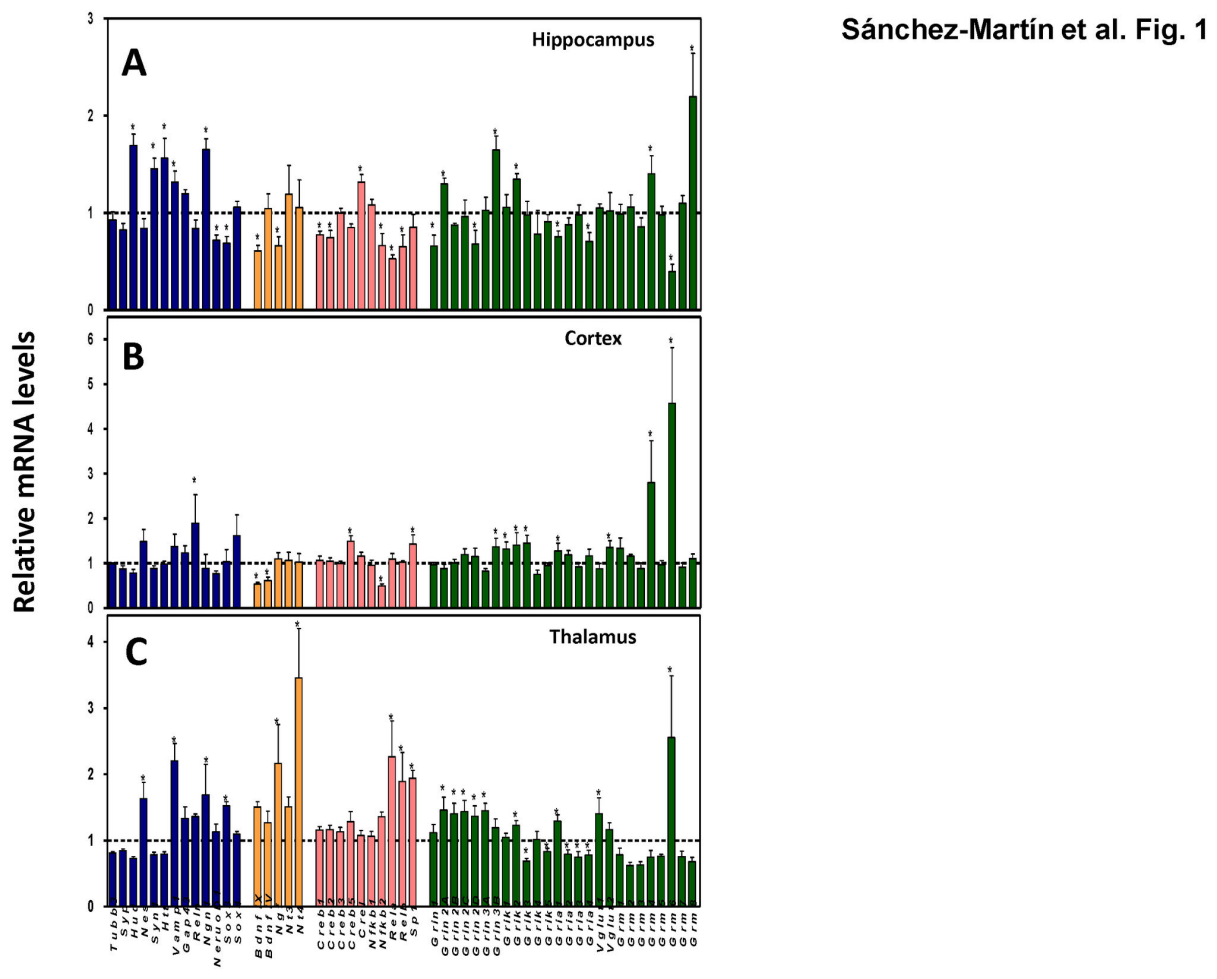
Expression patterns of genes altered in mice brain after 0 or 3 ppm of gestational Pb exposure. (**A**), Hippocampus; (**B**), cortex; and (**C**), thalamus at PND60 were dissected from 3 male mice in each group. Gene expression as determined from qRT-PCR levels was normalized to *Gapdh* expression in each condition and expressed relative to the corresponding level in controls with no Pb treatment. Tissues from each mouse were processed individually and the data shown represents the mean ± SEM of the three mice. (*) p< 0.05.

We used quantitative real-time RT-PCR (qRT-PCR) to measure the expression of several neural markers implicated in vesicle release (*Syn1*, *Vamp1*, and *Syp*), neurite and axon growth (*Tubb3*, *Nes*, and *Gap43*), axon maturation and synaptogenesis (*Reln*), mRNA stabilization (*Hud*), BDNF vesicle transport (*Htt*), and transcription factors involved in neuronal differentiation (*Ngn1*, *NeuroD1*, *Sox3*, and *Sox4*) (Figure 1, *blue bars*). We also examined the expression of the neurotrophins *Bdnf*, *Ngf*, *Nt3* and *Nt4* (Figure 1, *orange bars*), which are produced and secreted by neural and glial cells and are implicated in cell proliferation, maturation survival, axonal outgrowth, and neural plasticity [38]. We included transcription factors with key roles in the CNS, such as Sp1 and the members of the CREB and NF-κβ families (Figure 1, *pink bars*), and glutamate-related genes (Figure 1, *green bars*), including the different subunits of the glutamate receptors *Grin1*, *Grin2A*-*D*, and *Grin3A*-*B*, for the glutamate N-methyl-D-aspartate (NMDA) receptors, *Grik1*-*5* for the kainate receptors, *Gria1*-*4* for α-amino-3-hydroxy-5-methyl-4-isoxazolepropionic acid (AMPA) receptors, *Grm1*-*8* for the metabotropic receptors, and *Vglut1*-*2* for the vesicular glutamate transporters. Of the three tissues tested, hippocampus showed more Pb-induced gene expression changes than the others. The neural markers *Hud*, *Syn1*, *Htt*, *Vamp1*, and *Ngn1*, the member of the NF-κβ family *crel*, and the glutamate-related genes *Grin2A*, *Grin3B*, *Grik2*, *Grm4*, and *Grm8* were up-regulated, while the neural markers *NeuroD1* and *Sox3*, the neurotrophins *Bdnf* exon IX and *Ngf*, the transcription factor *Creb1* and *Creb2*, the members of the NF-κβ family *Nfκb2*, *Rela*, and *Relb*, and the subunits for glutamate receptors *Grin1*, *Grin2D*, *Gria1*, *Gria4*, and *Grm6* were down-regulated (Figure 1A). *Bdnf* exon IX contains the coding region, whereas exon IV, a non-coding exon, is up-regulated through calcium-dependent neural activity [39,40]. In cortex and thalamus, Pb exposure led more often to up-regulation of gene expression than to down-regulation. In cortex, the neural marker *Reln*, the member of the CRE family *Creb5*, the transcription factor *Sp1*, and *Grin3B*, *Grik1*, *Grik2*, *Grik3*, *Gria1*, *Grm4* and *Grm6* glutamate receptors were up-regulated, whereas exons IX and IV of the neurotrophin *Bdnf* and *Nfκb2* were down-regulated (Figure 1B). In thalamus, the neural markers *Nes*, *Vamp1*, *Ngn1*, and *Sox3*, the neurotrophins *Ngf* and *Nt4*, the members of the NF-κβ family *Rela* and *Relb*, the transcription factor *Sp1*, and glutamate-related genes *Grin2A*, *Grin2B*, *Grin2C*, *Grin2D*, *Grin3A*, *Grik2*, *Gria1*, *Vglut1*, and *Grm6* were up-regulated while the for glutamate receptor subunits *Grik3*, *Grik5*, *Gria2*, *Gria3*, and *Gria4* were down-regulated (Figure 1C). These results, observed 2 months after the time of exposure, support the conclusion that early-life exposure to Pb may produce irreversible changes that extend to adult life, producing alterations in the expression pattern of the genes analyzed.

### Neural differentiation of mouse ESC produces glutamatergic neurons

To identify molecular changes taking place during embryogenesis and neurodevelopment as a consequence of extended Pb exposure we used an *in vitro* model of neural differentiation of mouse ESCs. Unlike other toxicological models of primary neuron cultures or neuronal cell lines, such as PC12 or SHSY5H cells, developmental effects of Pb can be better studied in these cells because addition of Pb to the culture medium during the differentiation process allows us to determine whether changes that take place *in vivo* during gestational Pb-exposure occur also in Pb-treated neurons differentiated *in vitro*. 

**Figure 2:**
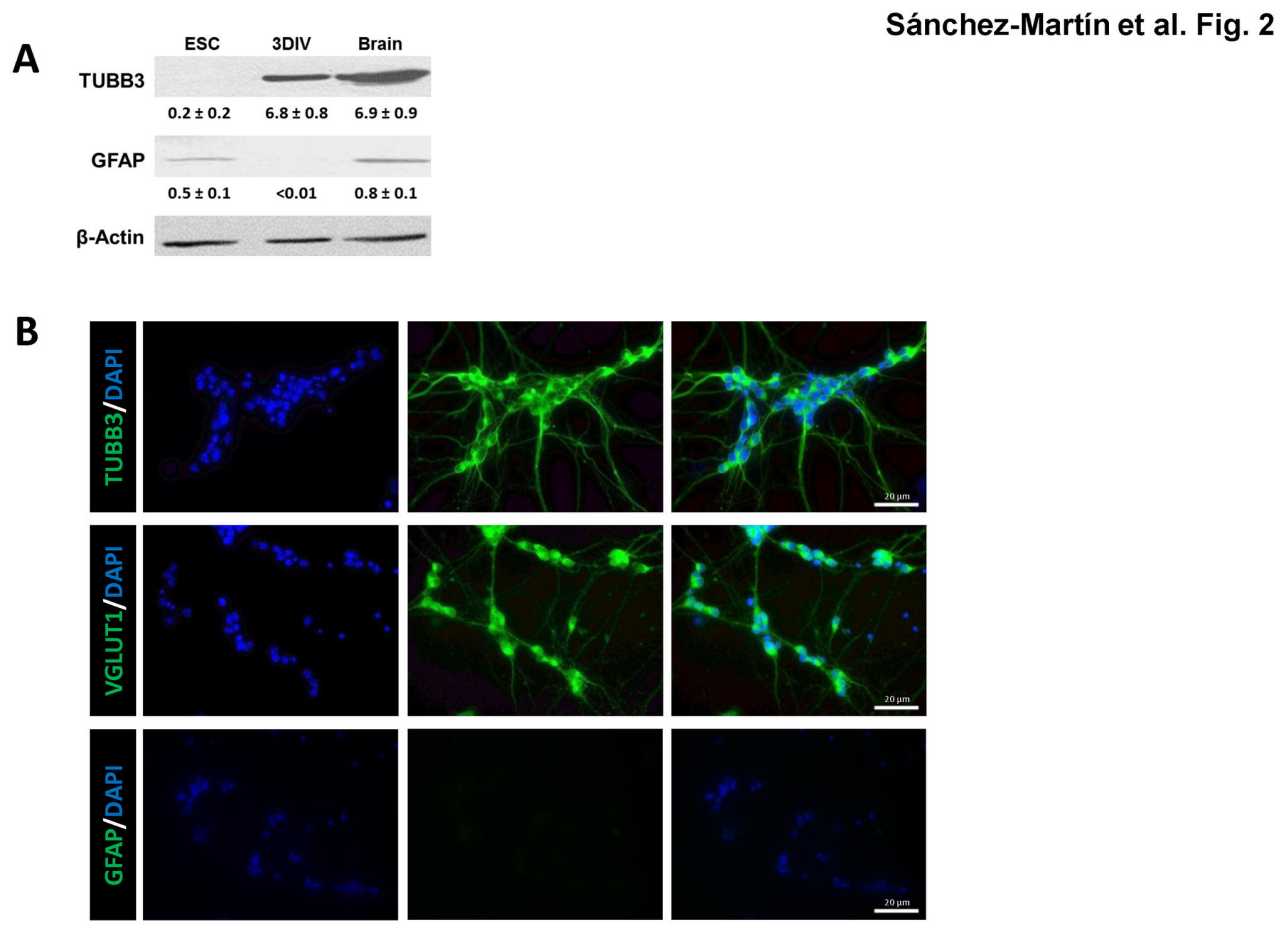
Expression of TUBB3, VGLUT1, and GFAP proteins. (**A**) Western immunoblot analysis of the neural marker TUBB3 and the glial marker GFAP. Representative western blots are shown for mouse embryonic stem cells (mESC), for neurons obtained from mESC neural differentiation 3 days after CA disaggregation (3DIV Neuron), and for mouse brain. Experiments were performed in three individual cultures and the mean ± SEM expression quantified relative to β-actin and shown below each lane. (**B**) Fluorescence detection of TUBB3, GFAP, and VGLUT1 proteins were carried out in neurons obtained from mESC neural differentiation 3 days after CA disaggregation. The third panel shows the merge for each pair of proteins.

We followed protocols previously established by others to induce differentiation of C57BL/6-C2 mouse ESC along neuronal lineages [29]. To verify the extent of neuronal differentiation and determine the level of contamination with glial cells, we used western immunoblot analysis for the neural marker TUBB3 and the glial marker GFAP. After 3 days *in vitro* (3-DIV) following cellular aggregate disaggregation, immunoblot analyses showed that 3-DIV cells expressed high levels of TUBB3 and no detectable GFAP (Figure 2A). As a positive control, a total protein extract from mouse brain showed high levels of both proteins (Figure 2A). Immunofluorescence analyses verified that most of the cells expressed TUBB3 and that there were no detectable cells positive for GFAP. Furthermore, immunofluorescence analyses showed that our protocol produced glutamatergic neurons, as demonstrated by the expression of VGLUT1 (Figure 2B), a marker of glutamatergic neurons [41,42]. Immunofluorescence quantification indicated that 85 % of the cells were TUBB3 and VGLUT1 positive (Figure S1). In addition, 9% of the cells in the culture were positive for active caspase-3, an indication of apoptosis (see below).

**Figure 3:**
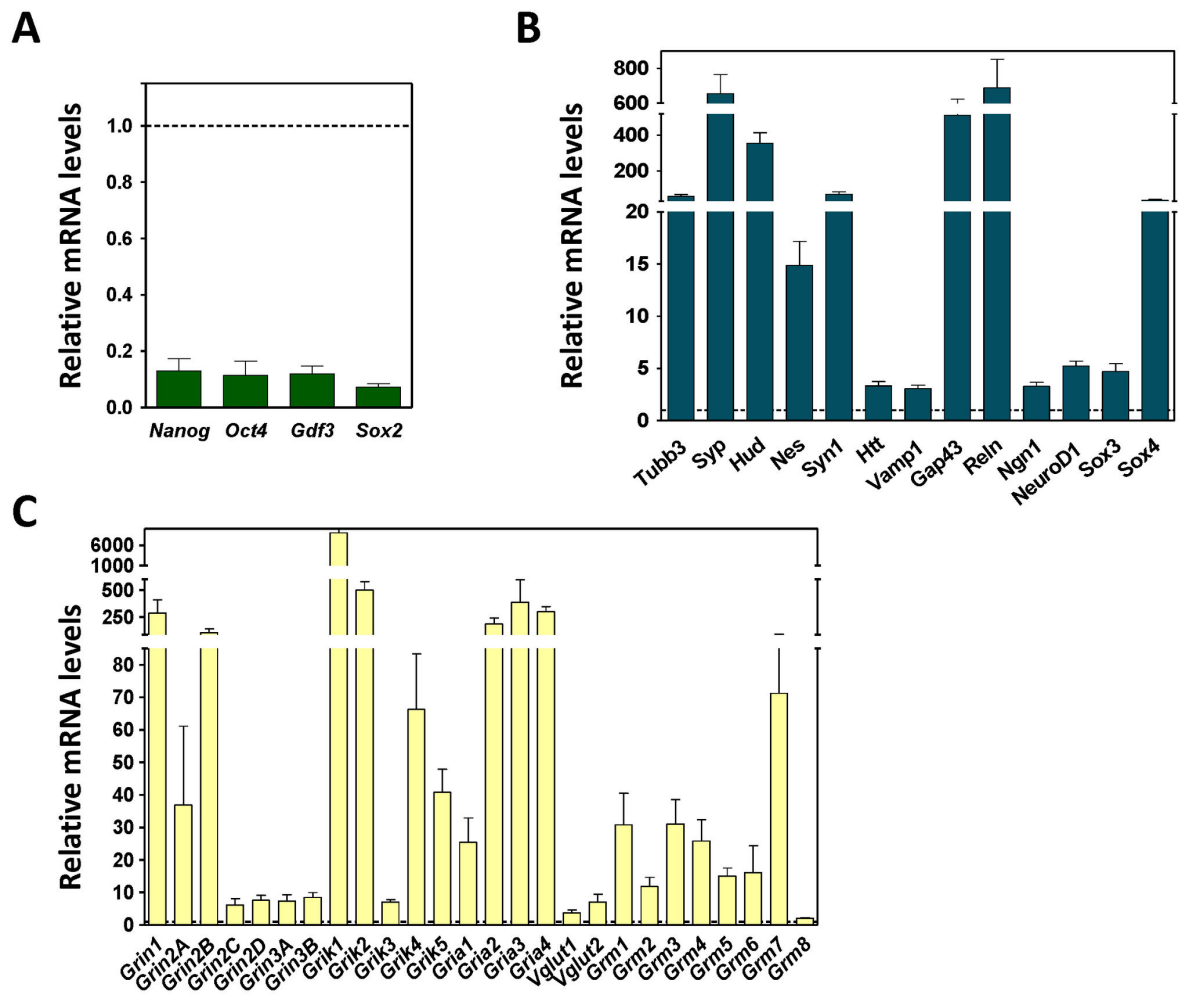
Gene expression pattern of neurons obtained from mESC neural differentiation 3 days after CA disaggregation. Total RNA was isolated from neurons obtained from mESC neural differentiation 3 days after CA disaggregation. Gene expression was normalized to *Gapdh* expression. The oligonucleotides used for amplification are described in Table S1. Gene expression was analyzed by qRT-PCR for, (**A**), pluripotency markers Nanog, Oct4, Sox2, Gdf3; (**B**), for neural markers; and (**C**), for glutamate receptors subunits and vesicular glutamate transporters. The data shown are the mean ± SEM from three independent determinations.

To further characterize the neural differentiation process of the ESC, we analyzed the expression of pluripotency and neural markers by qRT-PCR. Compared to undifferentiated ESC, the pluripotency genes, *Nanog*, *Oct4*, *Gdf3*, and *Sox2* were significantly down-regulated (Figure 3A). Among the neural markers analyzed there were genes involved in vesicle release (*Syn1*, *Vamp1*, and *Syp*), axon growth and maturation and synaptogenesis (*Tubb3*, *Nes*, *Gap43*, and *Reln*), transcription factors (*Ngn1*, *NeuroD1*, *Sox3*, and *Sox4*), BDNF vesicle transport (*Htt*), and mRNA stabilization (*Hud*). All the neural genes tested were significantly up-regulated, although to different extents (Figure 3B). In addition, we used qRT-PCR to assay for the subunits of NMDA, kainate, AMPA, and metabotropic receptors for glutamate, and for the glutamate transporters *Vglut1* and *Vglut2*. As for the neural markers, all the glutamate-related genes were up-regulated relative to their expression in ESC (Figure 3C), supporting the view that the ES cells had differentiated into a pure culture of glutamatergic neurons.

### Lead exposure diminishes cell numbers in cellular aggregates and changes gene expression pattern during ESC neural differentiation

**Figure 4:**
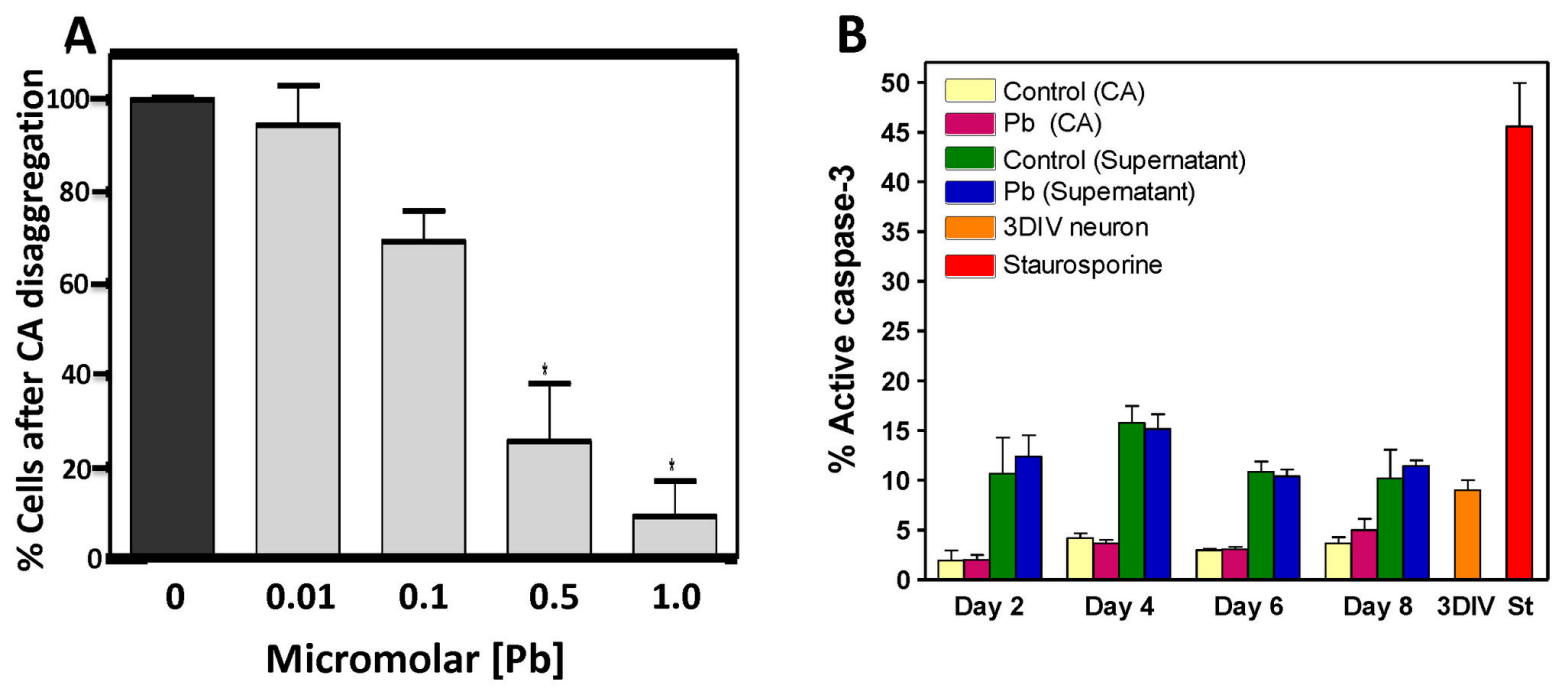
Effect of Pb in cellular aggregate formation. mESC were incubated with the indicated Pb concentrations during the complete process of CA formation. (**A**) After CA disaggregation, cell numbers were recorded. (**B**) Active caspase-3 determinations. Cells were treated with 0 (control) or 0.1 µMPb (Pb) during the 8 days of CA formation process. CAs were disaggregated every two days and active caspase-3 was measured in the cultures (Control, *yellow* *bars*; Pb, *pink* *bars*). The supernatant was taken every two days and active caspase-3 was measured in the cells in suspension (Control, *green* *bars*; Pb, *blue* *bars*). Neurons obtained from mESC neural differentiation 3 days after CA disaggregation were also analyzed (3DIV, *orange* *bar*). Staurosporine (St, red bars) was used as a positive control of active caspase-3 induced apoptosis. The experiments were performed in three independent cultures. (*) p< 0.05.

Treatment of cultured rat embryonic hippocampi neurons with micromolar Pb concentrations has been shown to decrease the levels of presynaptic vesicular proteins implicated in neurotransmitter released, CREB phosphorylation, and BDNF signaling [25,26]. In order to verify these results in our experiments and mimic gestational Pb exposure *in vitro*, we treated differentiating ES cells with Pb acetate for the length of the neuronal differentiation process and until day 3 *in vitro*. At 1 µM Pb the number of cells resulting from CA disaggregation was reduced by more than 90% relative to control untreated cells (Figure 4A). The effect of Pb treatment was clearly concentration dependent, reducing survival by 30 and 6% of control after treatment with 0.1 and 0.01 µM Pb, respectively (Figure 4A). We chose 0.1 µM as the concentration for all subsequent experiments.

Pb toxicity has been related to cell death by apoptosis [43]. To determine whether the loss of cells observed after CA disaggregation was caused by apoptosis, we measured active caspase-3 expression at different time points during CA formation. Interestingly, we found no difference in active caspase-3 levels at any point between control and Pb-treated cells during the whole process of CA formation, neither in the supernatant nor in the CA fraction (Figure 4B), compared to staurosporine as a positive control. This result indicates that loss of cells from the cellular aggregates is unlikely to result from apoptosis and more likely to be due to Pb interference with the competence of the cells to aggregate.

**Figure 5:**
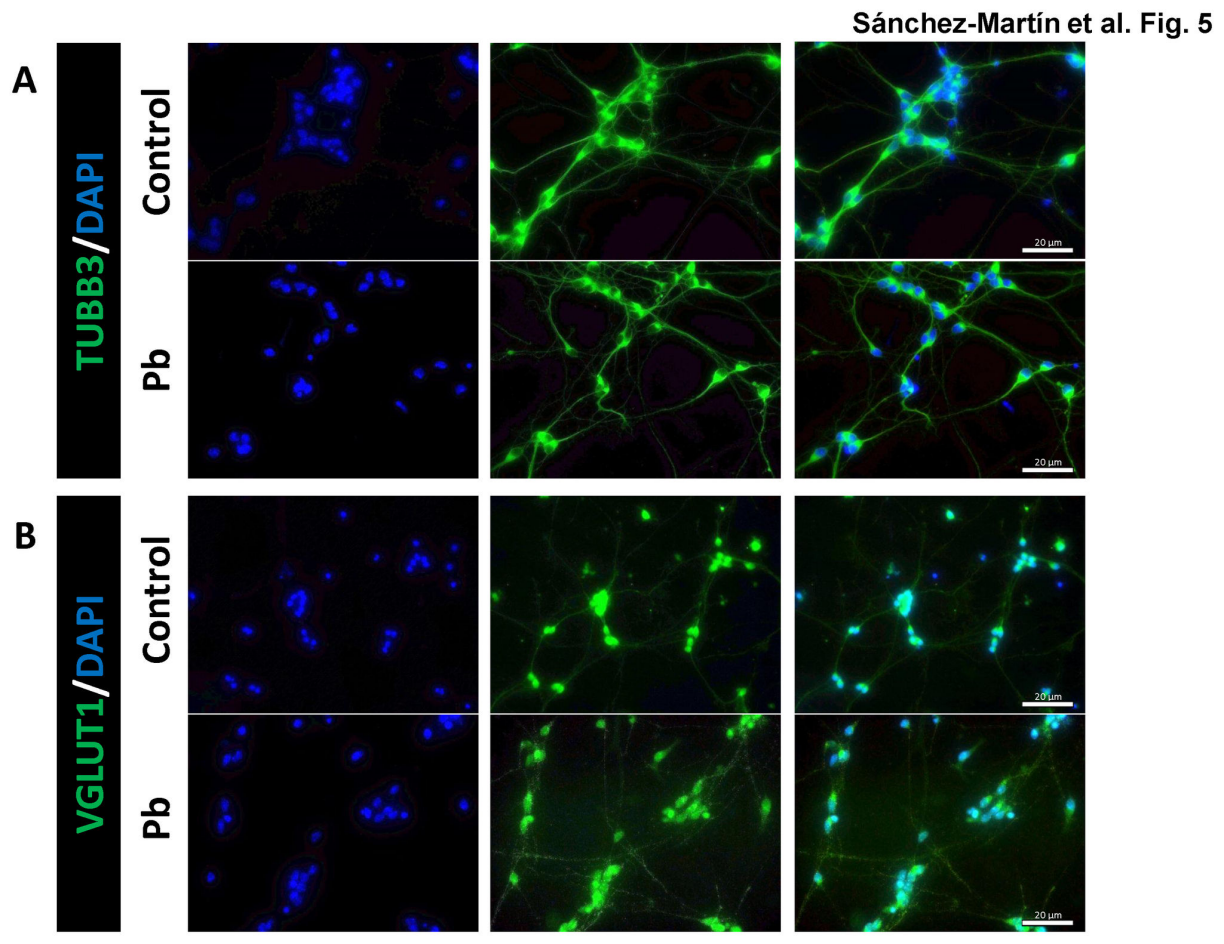
Immunofluorescence detection of TUBB3 (A) and VGLUT1 (B). Neurons obtained from mESC neural differentiation 3 days after CA disaggregation were untreated (control) or treated with 0.1 µM (Pb) during the whole differentiation process. The third panel shows the merge for each pair of proteins. The experiment shown is representative of three independent cultures.

**Figure 6:**
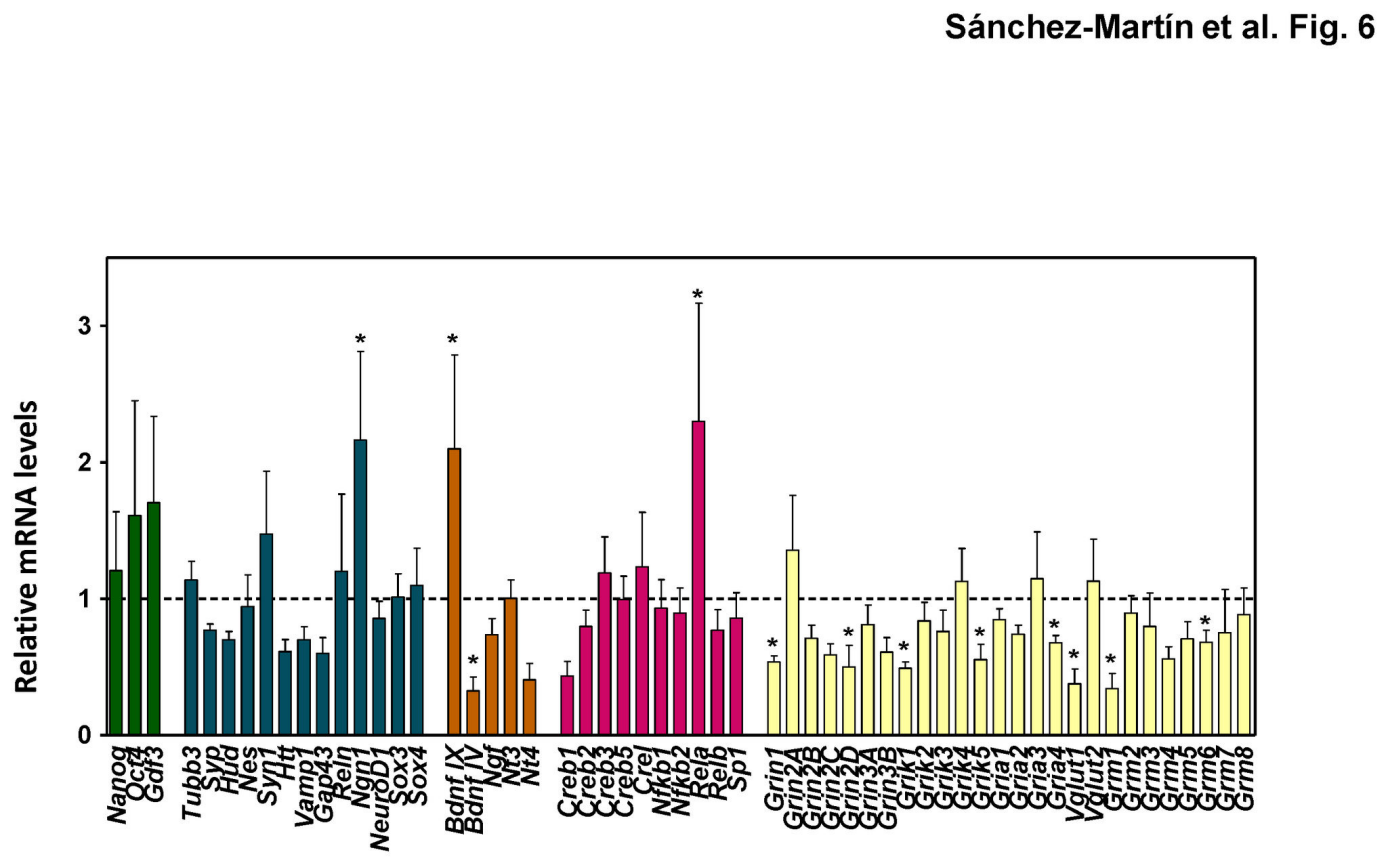
Expression patterns of genes altered in ESC-derived neurons after Pb treatment. mESC were untreated or treated with 0.1 µM of Pb during the whole neural differentiation process. Total RNA was isolated from neurons obtained from mESC neural differentiation 3 days after CA disaggregation. Gene expression was normalized to *Gapdh* expression in each condition and expressed relative to the corresponding level in untreated controls. *Green* *bars* represent pluripotency markers; *blue* *bars*, neural markers; *orange* *bars*, neurotrophins; *pink* *bars*, transcription factors; and *yellow* *bars*, glutamate receptors subunits and vesicular glutamate transporters. The data shown are the mean ± SEM of three independent replicates. (*) p< 0.05.

The neurons resulting from Pb treatment of ESC during the differentiation process are TUBB3 and VGLUT1 positive, morphologically indistinguishable from untreated control neurons, as determined by immunofluorescence analysis (Figure 5A and 5B). Expression of the pluripotency genes *Nanog*, *Oct4*, *Gdf3*, and *Sox2* was slightly up-regulated in these neurons, but not significantly different from their expression in untreated control neurons (Figure 6, *green bars*). The neural marker *Ngn1* was up-regulated after Pb treatment, whereas *Syp*, *Htt*, *Gap43*, and *Reln* were marginally down-regulated in the presence of Pb, although the difference was not statistically significant (Figure 6, *blue bars*). We also examined the differentiated neurons for expression of the neurotrophins and the transcription factors analyzed in the mouse brain tissues. *Bdnf* exon IV was down-regulated in cells treated with 0.1 µM Pb, whereas *Bdnf* exon IX was up-regulated (Figure 6, *orange bars*). *Creb1* was slightly down-regulated, with no significant difference to their expression in control neurons, and *Rela* was up-regulated by Pb. The rest of the genes analyzed did not change their expression (Figure 6, *pink bars*). To complete the analysis, we measured the expression of subunits for NMDA, kainate, AMPA, and metabotropic receptors for glutamate, and for the glutamate transporters *Vglut1* and *Vglut2*. Pb down-regulated *Grin1* and *Grin2D* of the NMDA receptor subunits, *Grik1* and *Grik5* of the kainate receptor subunits, *Gria4* of the AMPA receptor subunits, *Grm1* and *Grm6* of the metabotropic receptor, and *Vglut1* of the glutamate transporters. *Grin2A*, *Grin2B*, *Grin2C*, *Grin3B*, *Gria2*, and *Grm4* were marginally different from their expression in control neurons (Figure 6, *yellow bars*). These results show that Pb exhibits a repressive effect on the expression of glutamate receptors, leading to the conclusion that Pb treatment during neural differentiation process of ESC *in vitro* causes changes in gene expression patterns that affect multiple neuronal transport processes, including calcium signaling pathways.

**Figure 7:**
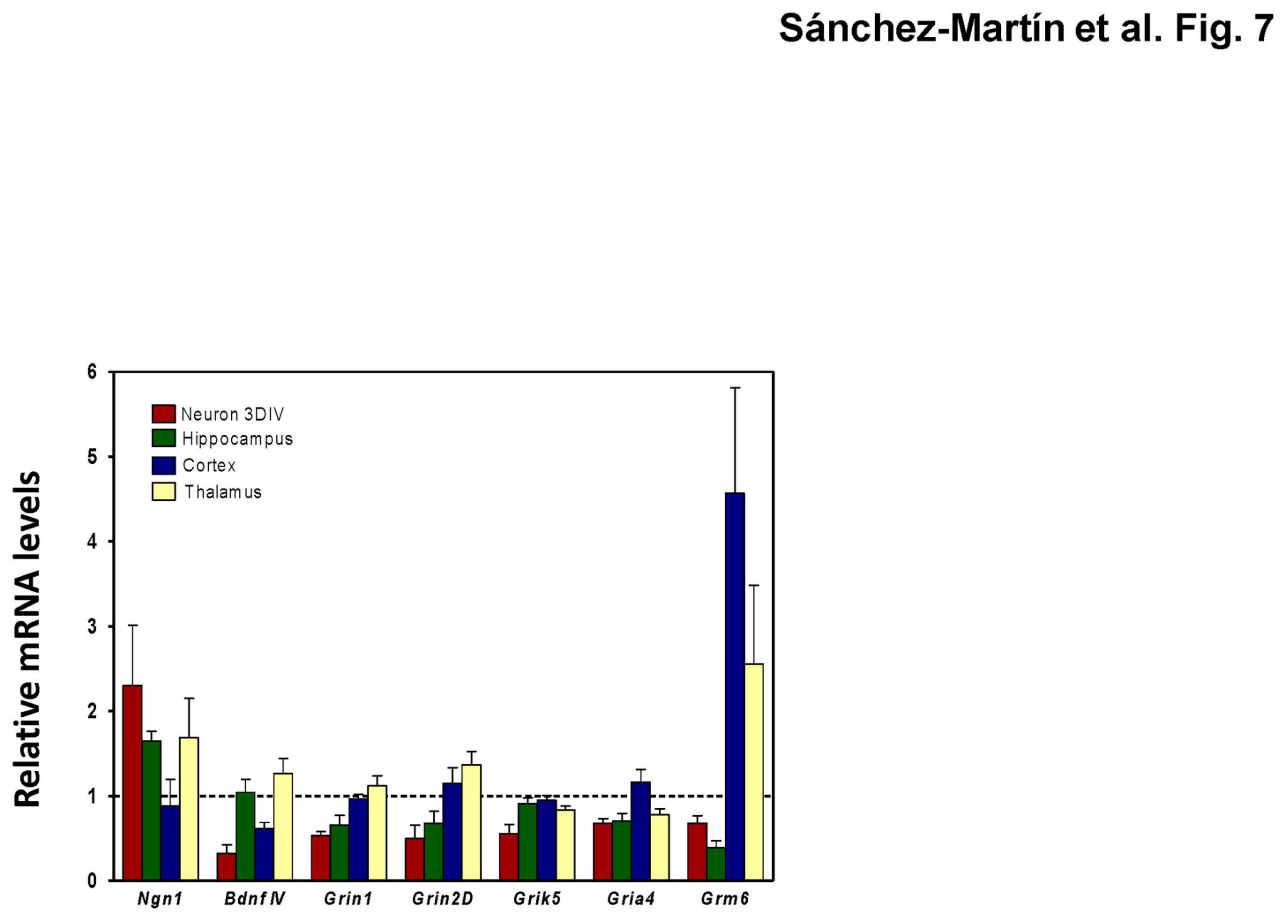
Expression patterns of genes with similar alterations in neural differentiated neurons treated with 0.1 µMPb and in mouse brain gestationally exposed to 3 ppm Pb. Neurons were treated with 0.1 µM Pb or left untreated for the length of the neural differentiation process. Mouse tissues were the same as in Figure 1. Gene expression was normalized to *Gapdh* expression and expressed relative to the corresponding levels in untreated or unexposed controls with no Pb treatment. The data shown are the mean ± SEM of three independent determinations.

A side-by-side comparison of Pb effects on gene expression in ESC-derived 3-DIV neurons and brain tissues reveals that more frequently repression is the consequence of Pb treatment in both cases. Thus, Pb up-regulates *Ngn1 in vitro* and in hippocampus, while it down-regulates the glutamate receptor subunits *Grin1*, *Grin2D*, *Gria4*, and *Grm6*. *Ngn1* is also up-regulated by Pb in the thalamus. *Bdnf* is down-regulated *in vitro* and in the cortex and the glutamate receptor subunits *Grm3* and *Grm4* subunits are down-regulated *in vitro* and in thalamus (Figure 7). These similarities in gene expression responses between mouse brain tissues gestationally exposed to Pb on the one hand, and ESC-derived neurons differentiated in the presence of Pb, on the other, support the concept that this model of toxic exposure during neural differentiation may be useful to analyze the mechanisms of toxicity by lead and other environmental neurotoxicants.

## Discussion

In this study we have found that mice exposed to Pb *in utero* show gene expression changes in adult life and that many of these changes can also be found in Pb-treated neurons differentiated *in vitro* from mouse ES cells. We followed the expression of genes coding for neural markers, neurotrophins, transcription factors, and glutamate-receptors at postnatal day 60 in three different brain regions, namely hippocampus, cortex, and thalamus. Among the three brain areas analyzed, hippocampus was the one to show more Pb-induced gene expression changes for the gene tested (*Hud*, *Syn1*, *Htt*, *Vamp1, Ngn1*, *NeuroD1*, *Sox3*, *Bdnf* exon IX, *Ngf*, *Creb1*, *Creb2*, *Crel*, *NFκβ2*, *Rela*, *Relb*, *Grin1*, *Grin2A*, *Grin2D*, *Grin3B*, *Grik2*, *Gria1*, *Gria4*, *Grm4*, *Grm6*, and *Grm8*), followed by thalamus (*Nes*, *Vamp1*, *Ngn1*, *Sox3*, *Ngf*, *Nt4*, *Rela*, *Relb*, *Sp1*, *Grin2A*, *Grin2B*, *Grin2C*, *Grin2D*, *Grin3A*, *Grik2*, *Grik3*, *Grik5*, *Gria1*, *Gria2*, *Gria3*, *Gria4*, *Vglut1*, and *Grm6*) and cortex (*Reln*, *Bdnf* exon IX and IV, *Creb5*, *NFκβ2*, *Sp1*, *Grin3B*, *Grik1*, *Grik2*, *Grik3*, *Gria1*, *Grm4*, and *Grm6*). After a side-by-side comparison of the effects of Pb on the gene expression in ESC-derived neurons and brain tissues, we observed that *Ngn1* was up-regulated and *Grin1*, *Grin2D* and *Grm6* were down-regulated *in vitro* and in hippocampus, being *Ngn1* also up-regulated in thalamus. *Bdnf* exon IV and *Grik5* were down-regulated in vitro and in cortex and thalamus, respectively. Finally, *Gria4* was repressed in vitro, in hippocampus, and thalamus. 

Recently, it has been described that reelin (RELN), implicated in axon growth and maturation, is decreased in zebrafish telencephalon [44] when exposed to Pb during development. Although we did observe a slightly down-regulation in the *in vitro* model, *Reln* was up-regulated in the cortex after gestational exposure to Pb, without any alteration in the other brain tissues analyzed. Decreases in synaptophysin (SYP) phosphorylation as a result of Pb exposure have been described recently in hippocampal primary cultures, suggesting that this disturbance may produce impairment in vesicular release in synapses [26,27]. The same study found changes in the phosphorylation status of huntingtin (HTT), which acts as a transcription factor and a regulator of BDNF vesicle transport in physiological conditions, and when mutated, is responsible for Huntington’s disease [45]. None of these genes, however, were affected by gestational Pb exposure in our studies, being marginally down-regulated in the 3-DIV neurons treated with Pb. In contrast, mRNA levels for the transcription factor gene neurogenin 1 (*Ngn1*), implicated in neural differentiation programs and cell fate [46], are significantly elevated in both our *in vitro* and *in vivo* models. Although we do not detect any morphological alterations when the cells are treated with Pb, our gene expression data suggest that the differentiation process may be compromised.

The ionotropic receptors NMDA, kainate, and AMPA, and the G-protein-coupled metabotropic receptor for glutamate are involved in excitatory transmission with important effects on synaptic plasticity that are implicated in neuronal processes such as learning and memory [47]. Recently, chronic exposure to Pb of primary hippocampal neurons has been shown to cause modifications on NMDA receptor composition as a result of decreases in the expression of GRIN1, GRIN2A, and GRIN2B [23,24]. Our results suggest that Pb alters the expression of the different glutamate receptors subunits and that this effect may cause an imbalance of calcium homeostasis responsible for the impairment of other signaling pathways. In our *in vivo* studies, *Grin2A* was up-regulated in hippocampus and thalamus of mice treated gestationally with Pb, but we did not observe any expression changes in cortex or in cultures of glutamatergic neurons. Additionally, *Grin1* was down-regulated in hippocampus and ESC-derived neurons exposed to Pb. Calcium conductance increases when the kainate receptors contain the *Grik5* subunit, while when they contain *Grik1* and *Grik2* they are less permeable to calcium [48,49]. Although there were more alterations in gene expression in other subunits of NMDA, kainate, AMPA, and metabotropic receptors by Pb in both models, Grik5 was down-regulated in both ESC-derived neurons treated with Pb and in thalamus, and Grik2 was up-regulated in cortex and thalamus of mice treated gestationally with Pb. If as a consequence of Pb treatment the glutamate receptors are reorganized, their functional properties may be sufficiently affected as to disrupt calcium homeostasis and signaling. 

Calcium signaling through the glutamate receptor has been shown to increase the level of CREB-dependent *Bdnf* transcripts containing exon IV [50], an effect that is down-regulated by Pb without affecting the expression of other non-coding exons [26]. In our experiments, *Bdnf* exon IX transcripts are repressed after Pb treatment in both hippocampus and cortex, being not affected in thalamus and increased in the neurons obtained from neural differentiation of mESCs. These discrepancies suggest that other non-coding exon(s) may play a role in the maintenance of *Bdnf* expression. The transcripts of the coding exon IV are down-regulated in cortex and our *in vitro* model, and not affected in hippocampus and thalamus. BDNF expression is highly controlled by different regulatory mechanisms, including epigenetic mechanisms, and mitogen-activated protein kinases, phosphoinositide-3 kinase and phospholipase-γ pathways [51]. As the neurotrophins are implicated in cell survival, differentiation, axon growth and guidance, synapses formation, and memory formation, the outcomes observed after Pb treatment provide a putative mechanism by which this metal may induce long-term potentiation (LTP) and spatial memory impairments. The role played by neurotrophins in such physiological events has recently been reinforced by the findings that antibodies against BDNF and NT4 blocked long-term recognition memory in rats [52]. Interestingly, we find that *Bdnf* exon IV and *Grin1* are down-regulated in cortex and hippocampus of mice treated gestationally with Pb and in 3-DIV neurons exposed to Pb during the neural differentiation process, suggesting that these changes may extend into adulthood, beyond the time when the organism was exposed to the agent.

Pb toxicity has been associated with apoptosis [43] and shown to induce an increase of caspase-3 cleavage and a decrease of intracellular glutathione levels in human SH-SY5Y neuroblastoma cells [53]. In our hands, however, 0.1 µM Pb reduced cell numbers after CA disaggregation without inducing concomitant cell death directly and without activating caspase-3. These results are remarkably similar to those described for low-concentration, long-term treatment of neural stem cells derived from E15 rat cortex, striatum, and ventral mesencephalon, and mouse bone marrow-mesenchymal stem cells, which show that cell proliferation is slightly altered at low Pb concentration and considerably affected at high concentration [54,55]. We tested treatment of ESC with different Pb concentrations in the range of 0.01 to 0.1 µM for 10 days and we did not observe any reduction of cell proliferation (Figure S2). Other in vivo studies have also shown discrepancies on casape-3 activation due to long-term exposure, suggesting that Pb effects may be region-, time-, and concentration-specific [56,57].

It is paramount to determine the molecular mechanisms by which Pb produces neurotoxicity. Although the use of lead has been reduced in the last few decades, its non-biodegradable nature and ubiquitous presence make it an environmental agent of general concern. Developmental or early-life exposure to Pb may produce CNS disorders detectable later in adulthood. The theory of the fetal origin of adult disease states that during development, exposure to environmental agents such as heavy metals, or to stressful situations like poor nutrition, may adversely contribute to adult pathogenesis [58,59], possibly by reprogramming specific gene expression patterns. Early-life exposure to lead may produce persistent changes in the mechanisms that regulate gene expression, contributing to adult neurological pathologies. Specifically, children are more sensitive to Pb exposure because absorption is greater in early life that in adults [60] and during the gestational period, Pb crosses the placenta and blood-brain barrier reaching the developing fetal brain [61]. Reduction of gray matter and alteration of the myelin structure and important metabolites are among the remote effects that early-life exposure to chronic and low-dose Pb produce in the CNS of adults [13]. Among its many other adverse effects, Pb is implicated in hepatic, renal, vascular, reproductive, and nervous diseases [3,4,7,8]. Additionally, lead accumulates in bones and blood, increasing the body burden and extending exposure well beyond the time of direct contact. Our current studies have developed a suitable *in vitro* model that uses mouse ESC differentiation to recapitulate neural gestational exposure to lead, which may serve to characterize the molecular mechanisms of lead neurotoxicity.

## Supporting Information

Figure S1 **Quantification of TUBB3 and VGLUT1 positive cells.** After fluorescence detection of TUBB3 and VGLUT1 neurons obtained from mESC, the percentage of TUBB3 and VGLUT1 positive cells was calculated as the Number of immunopositive cells/Total number of cells x100. At least five micrographs from different assays were analyzed. (*) p<0.05.
(TIF)

Figure S2 **Effects of Pb in mouse ESC cell number.** Mouse ES cells were treated with the indicated concentration of Pb during ten days. Cell numbers were recorded every other day at the time that the cells were passaged. Cell numbers were normalized to the number of cells plated on day 0.
(TIF)

Table S1 **List of primers used for real-time RT-PCR.**
(PDF)
