# Supplementary figures and images for: Lead Induces Similar Gene Expression Changes in Brains of Gestationally Exposed Adult Mice and in Neurons Differentiated from Mouse Embryonic Stem Cells
Source: PLoS One. 2013 Nov 19;8(11):e80558. doi: 10.1371/journal.pone.0080558 (PMC3834098; doi:10.1371/journal.pone.0080558)

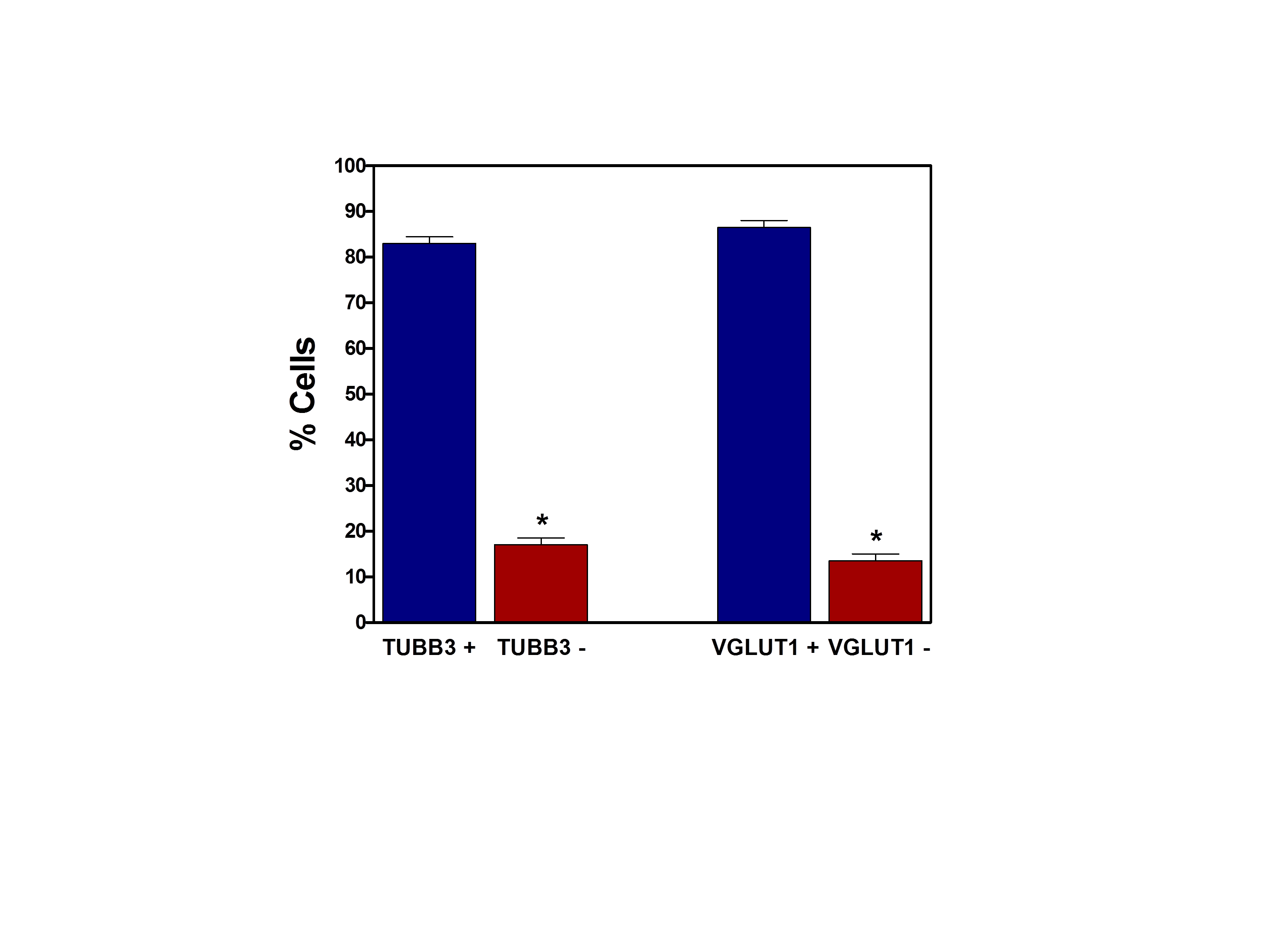

Supplement: Figure S1 — Quantification of TUBB3 and VGLUT1 positive cells. After fluorescence detection of TUBB3 and VGLUT1 neurons obtained from mESC, the percentage of TUBB3 and VGLUT1 positive cells was calculated as the Number of immunopositive cells/Total number of cells x100. At least five micrographs from different assays were analyzed. (*) p<0.05. (TIF) [file pone.0080558.s001.tif]

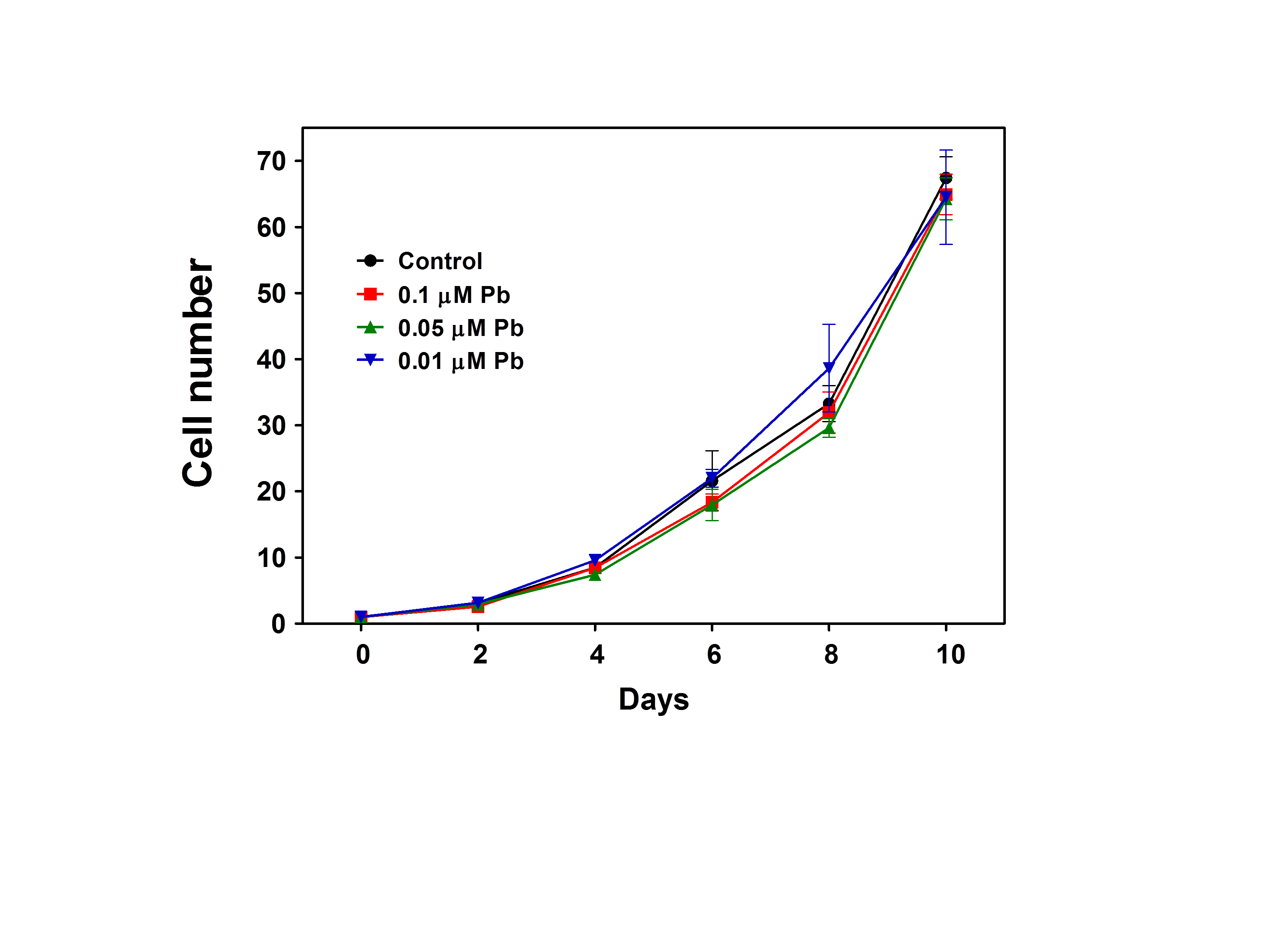

Supplement: Figure S2 — Effects of Pb in mouse ESC cell number. Mouse ES cells were treated with the indicated concentration of Pb during ten days. Cell numbers were recorded every other day at the time that the cells were passaged. Cell numbers were normalized to the number of cells plated on day 0. (TIF) [file pone.0080558.s002.tif]
