# Supplementary material for: Lead Induces Similar Gene Expression Changes in Brains of Gestationally Exposed Adult Mice and in Neurons Differentiated from Mouse Embryonic Stem Cells
Source: PLoS One. 2013 Nov 19;8(11):e80558. doi: 10.1371/journal.pone.0080558 (PMC3834098; doi:10.1371/journal.pone.0080558)
Supplement: Table S1 — List of primers used for real-time RT-PCR. (PDF) [file pone.0080558.s003.pdf]

Supplemental table S1 (I).Primer list.

Gene	Forward Sequence	Reverse Sequence
Nanog	5'-AGGGTCTGCTACTGAGATGCTCTG-3'	5'-CAACCACTGGTTTTTCTGCCACCG-3'
Oct4	5'-GGCGTTCTCTTTGGAAAGGTGTTC-3'	5'-CTCGAACCACATCCTTCTCT-3'
Gdf3	5'-ACCTTTCCAAGATGGCTCCT-3'	5'-CCTGAACCACAGACAGAGCA-3'
Sox2	5'-AAAGGAGAGAAGTTTGGAGCCCGA-3'	5'-GGGCGAAGTGCAATTGGGATGAAA-3'
Tubb3	5'-TTCTGGTGGACTTGGAACCTGGAA-3'	5'-TCACACTCTTTCCGCACGACATCT-3'
Syp	5'-CAGTGGGTCTTTGCCATCTT-3'	5'-CAAAGAATTCAGCCGAGGAG-3'
HuD	5'-CCAAAGGATGCAGAGAAAGC-3'	5'-GTGATGATGCGACCGTATTG-3'
Nes	5'-AGCAGAACCAGCTGCTCAGT-3'	5'-AGCTCTTCGGCAAGGTTGT-3'
Syn1	5'-GAGCAGATTGCCATGTCTGA-3'	5'-AGTTCCACGATGAGCTGCTT-3'
Htt	5'-ATCTCAGCCAGTCTGGTGCT-3'	5'-CCCACTGTTCTGGAGGTGTT-3'
Vamp1	5'-GAAGGGCAAAAACAAGGACA-3'	5'-GGGCAAGTTACAGCACATCA-3'
Gap43	5'-GGCTCTGCTACTACCGATGC-3'	5'-GGCTTGTTTAGGCTCCTCCT-3'
Reln	5'-CCATACTGTGGCCATGACTG-3'	5'-CACCTGGTTGTCCATGTGAG-3'
Ngn1	5'-GACACTGAGTCCTGGGGTTC-3'	5'-GTCGTGTGGAGCAGGTCTTT-3'
NeuroD1	5'-GGAGGAGGAGGATCAAAAGC-3'	5'-TGGGTCTTGGAGTAGCAAGG-3'
Sox3	5'-CACAACTCCGAGATCAGCAA-3'	5'-GTCCTTCTTGAGCAGCGTCT-3'
Sox4	5'-GAACGCCTTTATGGTGTGGT-3'	5'-TCAGGGTAGTCAGCCATGTG-3'
Bdnf (exon IX)	5'-TTGTTTTGTGCCGTTTACCA-3'	5'-GGTAAGAGAGCCAGCCACTG-3'
Bdnf (exon IV)	5'-ATTCACCGAGGAGAGGACTG-3'	5'-AGTCTTTGGTGGCCGATATG-3'
Ngf	5'-ACCACAGCCACAGACATCAA-3'	5'-GCCTTGACGAAGGTGTGAGT-3'
Nt3	5'-TGCAACGGACACAGAGCTAC-3'	5'-ACCCACAGGCTCTCACTGTC-3'

Supplemental table S1 (II).Primer list.

Gene	Forward Sequence	Reverse Sequence
Nt4	5’-AGTATGCGACGCAGTGAGTG-3’	5’-TTGCATTCTGAGAGCCAGTG-3’
Creb1	5’-GGTGCCAAGGATTGAAGAAG-3’	5’-CTGCCCACTGCTAGTTTGGT-3’
Creb2	5’-TCGATGCTCTGTTTCGAATG-3’	5’-GCCAATTGGGTTCACTGTCT-3’
Creb3	5’-ATGCTCTTCTCCGACTCCAA-3’	5’-CCTGAATACCTGCCCTGAAA-3’
Creb5	5’-AACCAAGCACAGGTTTCACC-3’	5’-TCTCATCTGGGTCCTCATCC-3’
Crel	5’-TGCTGGACATTGAAGACTGC-3’	5’-CCCCTGACACTTCCACAGTT-3’
NFκβ1	5’-CTGACCTGAGCCTTCTGGAC-3’	5’-GCAGGCTATTGCTCATCACA-3’
NFκβ2	5’-GAGAAGCCTGGTGGACACAT-3’	5’-GGTCTCAGGACCTTTCAGCA-3’
Rela	5’-TTCCTCAGCCATGGTACCTC-3’	5’-CCCCAAGTCTTCATCAGCAT-3’
Relb	5’-TGATCCACATGGAATCGAGA-3’	5’-CAGGAAGGGATATGGAAGCA-3’
Sp1	5’-CCAGTTATCACCAGGGCTGT-3’	5’-ACCCAGGGACAAAATTAGGG-3’
Grin1	5’-ACTCCCAACGACCACTTCAC-3’	5’-GTAGACGCGCATCATCTCAA-3’
Grin2a	5’-AGACCTTAGCAGGCCCTCTC-3’	5’-CTCTTGCTGTCCTCCAGACC-3’
Grin2b	5’-CCGCAGCACTATTGAGAACA-3’	5’-ATCCATGTGTAGCCGTAGCC-3’
Grin2c	5’-GCAGAACTTCCTGGACTTGC-3’	5’-CACAGCAGAACCTCCACTGA-3’
Grin2d	5’-CAGCTGCAGGTCATTTTTGA-3’	5’-GGATCTGCGCACTGACACTA-3’
Grin3a	5’-CAGAGGGATGAGCCAGAGTC-3’	5’-CTTCCACACGGTTCAGGTTT-3’
Grin3b	5’-CTACATCAAGGCGAGCTTCC-3’	5’-AGCTTGCAGTCCGCATCTAT-3’
Vglut1	5’-AGTCCTCCCTTGTTCCCAGT-3’	5’-GGTTGAACTGTCCCTCCAAA-3’
Vglut2	5’-ACCTGAGGCCTAGGAAGCTC-3’	5’-TCCCTGTTCTGGAAGTCACC-3’
Grik1	5’-TTAACCGAAACCGAACCTTG-3’	5’-TTCCAGCGAGTCTGAATGTG-3’

Supplemental table S1 (III).Primer list.

Gene	Forward Sequence	Reverse Sequence
Grik2	5’-GCTGTTCAGTCCATCTGCAA-3’	5’-AAGCGAATGAGACCAGTGCT-3’
Grik3	5’-ATGACAAAGGCCAGTGGAAC-3’	5’-AGAAGACACTGGGGTTGGTG-3’
Grik4	5’-AATGGGTTTCAGCAGATTGG-3’	5’-GTGGTTCCCCTTCAGCATTA-3’
Grik5	5’-AGTACGAGACCACGGACACC-3’	5’-CGAAGCGAAGGTACTGAAGG-3’
Gria1	5’-CTAGGCTGCCTGAACCTTTG-3’	5’-GGGAAGATTGAATGGAAGCA-3’
Gria2	5’-GACTTCAGGAGCAGGGACAG-3’	5’-TCGTTTCCCTTTCCTCCTTT-3’
Gria3	5’-CTCCTGATCCTCCCAATGAA-3’	5’-TCAGGAAAGCAGCAAGGTTT-3’
Gria4	5’-TTTGCAGGCAGATTGTCTTG-3’	5’-GGGGCTGGTGTTATGAAGAA-3’
Grm1	5’-TCATACGGAAAGGGGAAGTG-3’	5’-AAAAGGCGATGGCTATGATG-3’
Grm2	5’-CTAGAGGCCATGCTTTTTGC-3’	5’-CTGTGGGAGCATCACTGAGA-3’
Grm3	5’-GAAAGGACAAAGGCAGCAAG-3’	5’-GCTAAAAGAGCCCGTCACTG-3’
Grm4	5’-GGACACCTGCTCAAGGGATA-3’	5’-CTGATCTGGGGGATCTTGAA-3’
Grim5	5’-ACGAAGACCAACCGTATTGC-3’	5’-AGACTTCTCGGATGCTTGGA-3’
Grim6	5’-GTGGGAGTGATAGCGTGGTT-3’	5’-TGACCATGAGCAGGAGACTG-3’
Grim7	5’-AAATGGTGATGCTCCAGGAC-3’	5’-AGGGGTTCCCTTCTGTGTCT-3’
Grim8	5’-GCCACTGGACCAATCAACTT-3’	5’-GGGCAGAGTTCACAGGAGAG-3’
Gapdh	5’-ACCCTGTTGCTGTAGCCGTATTCA-3’	5’-TCAACAGCAACTCCCACTCTTCCA-3’
